# Dietary Nitrate-Rich Vegetables as Natural Modulators of Health: Mechanisms and Benefits in Ageing Populations

**DOI:** 10.3390/ijms27083461

**Published:** 2026-04-12

**Authors:** Natalia Kurhaluk, Renata Kołodziejska, Lyudmyla Buyun, Piotr Kamiński, Halina Tkaczenko

**Affiliations:** 1 Institute of Biology, Pomeranian University in Słupsk, Arciszewski St. 22B, 76-200 Slupsk, Poland; 2 Department of Medical Biology and Biochemistry, Faculty of Medicine, Collegium Medicum in Bydgoszcz, Nicolaus Copernicus University in Toruń, M. Karłowicz St. 24, 85-092 Bydgoszcz, Poland; renatak@cm.umk.pl (R.K.); piotr.kaminski@cm.umk.pl (P.K.); 3 Department of Tropical and Subtropical Plants, M.M. Gryshko National Botanic Garden, National Academy of Science of Ukraine, Sadovo-Botanichna St. 1, 01003 Kyiv, Ukraine; orchids.lyuda@gmail.com; 4 Department of Nature Conservation, Institute of Biological Sciences, University of Zielona Góra, Prof. Z. Szafran St. 1, 65-516 Zielona Gora, Poland

**Keywords:** phytochemical synergy, nitrate-responsive polyphenols, beetroot bioactives, antioxidant-nitric oxide interactions, plant-derived nitrite modulators, vascular phytochemical signaling

## Abstract

Nitrate-rich vegetables are increasingly recognised as a key subgroup of phytochemical-dense foods that have significant potential for preventing and managing chronic diseases. Although dietary nitrates were historically approached with caution due to concerns about nitrosamine formation, contemporary evidence highlights their beneficial effects on vascular, metabolic and cognitive functions. Ageing is characterised by endothelial dysfunction, impaired nitric oxide (NO) synthesis and increased oxidative stress, which elevates cardiovascular risk. In this context, nitrate-rich vegetables offer a natural way to restore NO bioavailability and support cardiometabolic health. This narrative review provides an integrative overview of nitrate-rich vegetables as sources of bioactive phytochemicals with therapeutic relevance. We summarise the biochemical pathways of nitrate and nitrite metabolism, including the enterosalivary nitrate–nitrite–NO cycle, the role of oral microbiota, and red blood cell-mediated nitrite reduction. Particular emphasis is placed on NOS-independent NO production, which becomes increasingly important with age, and on the synergistic interactions between dietary nitrates and other phytochemicals such as polyphenols, vitamin C, flavonoids and betalains. These compounds enhance NO stability, reduce oxidative stress, modulate inflammatory signalling and support mitochondrial function, thereby amplifying the health benefits of nitrate-rich vegetables. Beetroot, with its high nitrate content and distinctive antioxidant profile, is highlighted as a prime example. Clinical and mechanistic studies suggest that nitrate-rich vegetables may lower blood pressure, improve endothelial function and cerebral perfusion, enhance cognitive performance and muscle oxygenation, and increase exercise efficiency, particularly in older adults. Additional benefits include anti-inflammatory effects, modulation of platelet function and improvements in metabolic parameters, all of which are relevant to the prevention of chronic diseases such as hypertension, type 2 diabetes and atherosclerosis. While dietary nitrate is generally considered low-risk for healthy adults, caution is warranted in susceptible populations, such as infants and individuals with impaired renal function. Finally, significant research gaps remain, including the need for long-term, well-controlled trials and personalised strategies that account for variability in microbiota composition and nitrate metabolism between individuals.

## 1. Introduction

Over the past decade, there has been a growing body of scientific evidence supporting the role of plant-based dietary patterns in improving metabolic health and reducing the risk of chronic diseases. Plant foods are rich in phytochemicals, which are bioactive compounds that modulate metabolic, inflammatory and redox pathways, thereby contributing to physiological resilience and disease prevention [1]. There is consistent evidence that links well-balanced, minimally processed plant-based diets with a lower incidence of type 2 diabetes, improved glycaemic control and reduced cardiometabolic risk [2,3,4]. These benefits are attributed to the synergistic action of whole-food matrices rather than isolated nutrients. In this context, nitrate-rich vegetables, particularly leafy greens and beetroot, have emerged as a distinct category that is particularly relevant for ageing populations.

Dietary nitrates, which were previously viewed primarily from a toxicological perspective due to their potential role in *N*-nitrosocompound formation [5], are now recognised as essential elements of the nitrate-nitrite-nitric oxide (NO) pathway. The enterosalivary system regulates vascular tone, mitochondrial efficiency and cellular signalling, and acts as a complement to the classical L-arginine-NO synthase pathway, particularly in hypoxic or acidic conditions [6,7,8,9]. It is important to note that the physiological effects of dietary nitrate depend on the dose, the food source and the metabolic context [10]. Plant-derived nitrates are embedded in matrices rich in antioxidants and polyphenols, which can inhibit nitrosation reactions and enhance NO bioavailability [11].

Distinguishing between nitrate sources is also central to ongoing regulatory and technological debates. Although nitrates are commonly used in processed meats for antimicrobial and sensory purposes [12], concerns remain about their safety due to the formation of nitrosamines during processing. By contrast, nitrate-rich vegetables such as beetroot offer high nitrate levels alongside a variety of phytochemicals which may have synergistic antioxidant and cardioprotective properties [13,14]. However, some authors argue that safety is determined more by processing conditions and accompanying compounds than by the origin of the nitrates alone [15]. Population studies further highlight variability in nitrate intake across dietary patterns and regions [16], emphasising the importance of contextual interpretation.

Interest in dietary nitrate has increased with the recognition of NO as a multifunctional signalling molecule involved in vascular regulation, neurotransmission, inflammation, and neuroprotection—processes that decline with ageing [17]. Unlike pharmacological NO donors, dietary nitrate provides a more physiologically integrated approach, relying on endogenous and microbiome-mediated pathways to increase NO bioavailability. However, age-related changes to the oral microbiome may reduce the efficiency of converting nitrate to nitrite, placing dietary nitrate within a host–microbiome metabolic axis [18].

There is clinical evidence supporting the functional relevance of nitrate-rich foods. Interventions involving beetroot have been shown to reduce blood pressure in various populations, including pregnant women with hypertension, adults with elevated blood pressure and older individuals [19,20,21]. Interestingly, some of these antihypertensive effects appear to be independent of nitrate content, which suggests that there are synergistic interactions with other phytochemicals [22]. These findings highlight the multifunctional nature of nitrate-rich foods in modulating vascular and systemic physiology.

## 2. Search Strategy and Study Selection

This narrative review examines the role of dietary nitrate-rich vegetables as natural modulators of health, focusing on mechanisms and benefits relevant to ageing populations. Evidence from vascular biology, microbiome research, phytochemistry and clinical nutrition is integrated to explore how nitrate-rich vegetables and their associated phytochemicals influence nitric oxide (NO) bioavailability, vascular function and cognitive resilience. A structured literature search was conducted in PubMed, Scopus, Web of Science and Google Scholar, covering publications from 1990 to 2025. The search terms reflected the mechanistic and phytochemical focus of the review and included ‘phytochemical synergy’, ‘nitrate-responsive polyphenols’, ‘beetroot bioactives’, ‘antioxidant–nitric oxide interactions’, ‘plant-derived nitrite modulators’ and ‘vascular phytochemical signalling’. Boolean operators were used to combine terms relating to dietary nitrates, NO metabolism, vascular outcomes, cognitive ageing and phytochemical interactions. Only peer-reviewed articles published in English were included, with an emphasis placed on clinical trials, mechanistic studies and high-quality reviews. Additional relevant publications were identified through manual screening of reference lists and forward citation tracking. As this is a narrative review, predefined eligibility criteria, systematic screening procedures and quantitative synthesis were not involved. However, the search strategy and study selection rationale are reported transparently to ensure clarity and reproducibility.

## 3. Biochemical Pathways of Dietary Nitrates and Nitrites

### 3.1. Enterosalivary Nitrate Cycle

Absorption of dietary nitrate begins in the small intestine, where it enters the bloodstream via the intestinal mucosa, mainly from leafy vegetables. This process is highly efficient, with plasma nitrate levels increasing within 30–60 min of ingestion and bioavailability exceeding 90% due to minimal first-pass metabolism and rapid systemic distribution [23,24,25]. Around 25% of the nitrate in circulation is actively taken up by the salivary glands via the sialin transporter, leading to a 10–20-fold increase in salivary nitrate concentration compared to plasma [23,26]. This enterosalivary circulation, comprising intestinal absorption, systemic distribution, salivary concentration, and re-swallowing, maintains a circulating nitrate reservoir that supports NO production, particularly under hypoxic conditions where endogenous NO synthase (NOS) activity is limited [27,28].

In the oral cavity, nitrate is reduced to nitrite (NO_2_^−^) by bacteria that can reduce nitrates, which are mainly located on the dorsal surface of the tongue [26]. Since humans lack efficient endogenous nitrate reductases, this step is crucial for the nitrate-nitrite-NO pathway [29]. Under physiological conditions, approximately 5–7% of ingested nitrate is converted to nitrite; however, this process is strongly influenced by the composition of the oral microbiome [23,30]. Individuals with a higher abundance of nitrate-reducing bacteria (e.g., *Neisseria*, *Rothia*, *Actinomyces* and *Veillonella*) exhibit greater nitrite production [31,32]. Diet is a key factor, as a higher intake of nitrate-rich vegetables has been shown to increase salivary and plasma nitrite levels, as well as improving blood pressure profiles [33].

After being swallowed, nitrite enters the acidic environment of the stomach, where it is partially reduced to NO and other reactive nitrogen species in a non-enzymatic process. This contributes to the body’s antimicrobial defences and the regulation of gastric blood flow. The remaining nitrite is absorbed into the bloodstream and acts as a systemic NO reservoir, particularly in hypoxic and acidic conditions [34]. Disruption to the oral microbiome by antibacterial mouthwashes, such as chlorhexidine, significantly impairs nitrate reduction and decreases nitrite bioavailability, thereby attenuating the blood-pressure-lowering effects of dietary nitrate [29,31,33]. Thus, the enterosalivary nitrate cycle is an integrated host-microbiome system, where intestinal absorption, salivary concentration, microbial reduction and systemic recirculation collectively sustain NO bioavailability, supporting cardiometabolic homeostasis.

### 3.2. NOS-Independent Nitric Oxide Production

Recognising that NO can be generated independently of NOS has significantly expanded our understanding of vascular regulation and redox biology. Early studies showed that nitrite can be converted into NO in acidic and hypoxic environments, which are characteristic of ischaemic tissues. This challenges the idea that NO production relies solely on the oxygen-dependent L-arginine-NOS pathway [35]. Nitrite is now considered a circulating reservoir of NO, which can be reduced by haem proteins, xanthine oxidoreductase, and components of the mitochondrial electron transport chain. The reaction rate increases as oxygen tension declines [36,37,38]. This NOS-independent pathway therefore functions as a physiological ‘backup system’ that preserves NO signalling during hypoxia and metabolic stress. Emerging evidence indicates that this mechanism also responds to environmental and tissue-specific stimuli. For instance, activation of the transient receptor potential vanilloid 3 (TRPV3) channel in the skin can stimulate NO formation independently of NOS, thereby linking nitrite-derived NO to thermoregulation and cutaneous blood flow [39]. In cardiac tissue, nitrite-derived NO supports mitochondrial function and provides protection against ischaemia-reperfusion injury [40].

**Figure 1:**
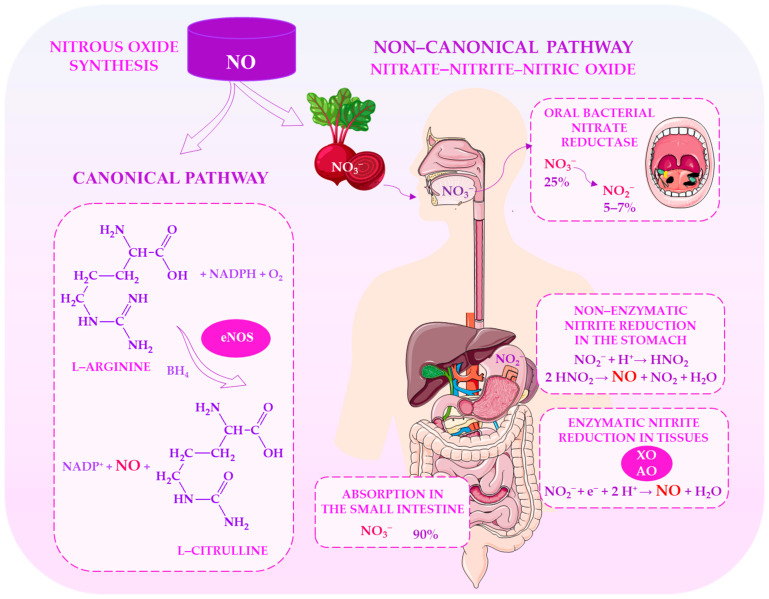
Conventional and alternative routes of nitric oxide production. Under normal physiological conditions, nitric oxide (NO) is primarily generated through the classical pathway mediated by nitric oxide synthases (NOS). In this process, L-arginine is enzymatically converted into NO and L-citrulline. However, during hypoxia, increased oxidative stress, or aging, the efficiency of this enzymatic pathway declines. An additional mechanism responsible for NO formation is the nitrate–nitrite–NO pathway, which becomes particularly important in environments characterized by low oxygen availability and acidic pH. In this pathway, dietary nitrates (NO_3_^−^), found in high amounts in vegetables such as beetroot, are first reduced to nitrites (NO_2_^−^) by oral microbiota. Subsequently, nitrites can be further converted to NO in the stomach and peripheral tissues through both non-enzymatic reactions and enzymatic processes involving xanthine oxidase and aldehyde oxidase. Image provided by Servier Medical Art (https://smart.servier.com, accessed on 1 March 2026), licensed under CC BY 4.0 (https://creativecommons.org/licenses/by/4.0/, accessed on 1 March 2026). Abbreviations: AO—aldehyde oxidase; HNO_2_—nitrous acid; NADP^+^—nicotinamide adenine dinucleotide phosphate (oxidized form); NADPH—nicotinamide adenine dinucleotide phosphate (reduced form); NO—nitric oxide; NO_2_—nitrogen dioxide; NO_2_^−^—nitrite; NO_3_^−^—nitrate; XO—xanthine oxidase.

Figure 1 shows the two main pathways of NO production. The first is the classical enzymatic route, in which NOS converts L-arginine to NO and L-citrulline. The second is the alternative nitrate-nitrite-NO pathway, which generates NO from dietary nitrates via oral microbiota and subsequent enzymatic and non-enzymatic reduction, particularly under hypoxic or oxidative stress conditions.

The physiological relevance of NOS-independent NO production is evident in studies of exercise and nutrition. Dietary nitrate is sequentially reduced to nitrite and then to NO. This enhances oxygen delivery, reduces the oxygen cost of submaximal exercise, and improves endurance performance. This is likely due to improved mitochondrial efficiency and muscle perfusion [41,42,43,44]. However, responses can vary between individuals [45,46]. Greater benefits are typically observed in less-trained individuals, whereas highly trained athletes often show limited responses due to their already optimised endogenous NO pathways [47,48,49]. Notably, whole-food nitrate sources appear to be more effective than isolated nitrate salts, potentially due to synergistic interactions with phytochemicals such as polyphenols and betalains [50].

At the biochemical level, nitrite acts as a redox-active intermediate that serves as both an oxidation product of NO and a substrate for its regeneration [51]. In the gastrointestinal tract, nitrite formed by oral bacteria can be further reduced to NO in an acidic environment, such as the stomach, supporting antimicrobial defence and the regulation of gastric blood flow [52]. Experimental studies demonstrate that nitrite-induced vasodilation is increased in mildly acidic conditions, which is consistent with its function as a conditionally activated NO donor [53]. This reduction is mediated by complementary systems, including deoxyhaemoglobin, deoxymyoglobin, xanthine oxidoreductase, and mitochondrial enzymes [54,55,56]. Dietary antioxidants (e.g., polyphenols and ascorbate) may further enhance this process and stabilise reactive nitrogen species [51].

The importance of this pathway increases with age. Reduced eNOS activity and availability of L-arginine, coupled with increased levels of endogenous inhibitors such as asymmetric dimethylarginine (ADMA), lead to impaired NO production and endothelial dysfunction [57]. Concurrently, elevated oxidative stress accelerates NO degradation [58]. In contrast, nitrite reduction is favoured under hypoxic and acidic conditions, which are more prevalent in ageing tissues. Consequently, older individuals may exhibit greater responsiveness to dietary nitrate interventions despite having lower baseline NO levels [59]. Indeed, nitrate-rich beetroot supplementation has consistently been shown to improve oxygen utilisation and cardiopulmonary efficiency in these populations [48,50].

**Figure 2:**
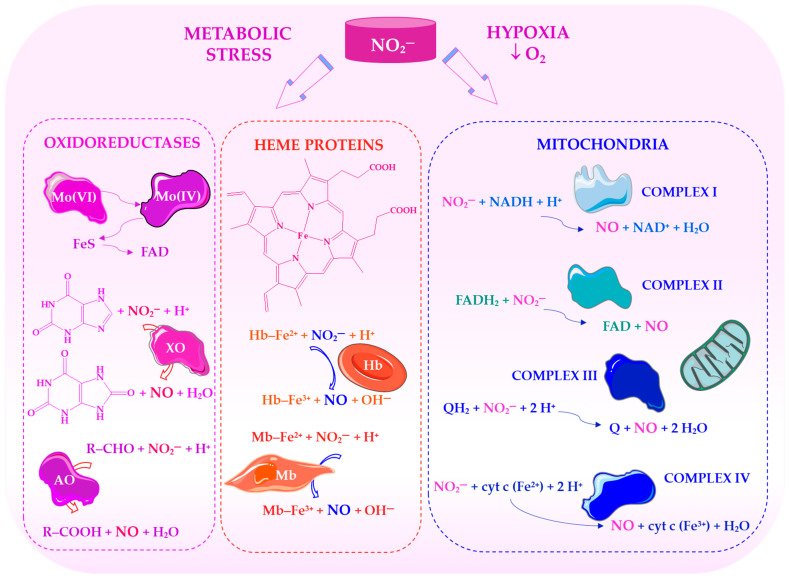
Nitrite as an alternative source of nitric oxide under hypoxic conditions. Nitrite (NO_2_^−^) acts as an important reservoir of nitric oxide (NO) in the body and can be reduced to NO by heme proteins, xanthine oxidoreductase, and components of the mitochondrial electron transport chain. This reduction becomes more efficient at low oxygen tension, when nitric oxide synthase (NOS) activity is limited. Consequently, the nitrate–nitrite–NO pathway functions as a physiological backup system that helps maintain NO signalling and vascular regulation during hypoxia and metabolic stress. Image provided by Servier Medical Art (https://smart.servier.com, accessed on 1 March 2026), licensed under CC BY 4.0 (https://creativecommons.org/licenses/by/4.0/, accessed on 1 March 2026). Abbreviations: AO—aldehyde oxidase; cyt c(Fe^2+^)—cytochrome c, reduced form (ferrous iron); cyt c(Fe^3+^)—cytochrome c, oxidized form (ferric iron); FAD—flavin adenine dinucleotide; FADH_2_—reduced flavin adenine dinucleotide; FeS—iron—sulfur cluster; Hb-Fe^2+^—deoxyhemoglobin (hemoglobin with ferrous iron); Hb-Fe^3+^—methemoglobin (hemoglobin with ferric iron); Mb-Fe^2+^—deoxymyoglobin (myoglobin with ferrous iron); Mb-Fe^3+^—metmyoglobin (myoglobin with ferric iron); NAD^+^—nicotinamide adenine dinucleotide (oxidized form); NADH—reduced nicotinamide adenine dinucleotide; NO—nitric oxide; NO_2_^−^—nitrite; Q—ubiquinone (coenzyme Q); QH_2_—ubiquinol (reduced coenzyme Q); XO—xanthine oxidoreductase.

Figure 2 shows how nitrite acts as an alternative source of NO under hypoxic conditions. It illustrates how nitrite is reduced to NO by haem proteins, xanthine oxidoreductase and mitochondrial enzymes. This supports NO signalling and vascular function when NOS activity is limited.

Mitochondria play a central role in this adaptive response (Figure 2). Under low-oxygen conditions, they can reduce nitrite to NO, thereby modulating respiration and protecting against ischaemia-reperfusion injury. NO inhibits cytochrome c oxidase reversibly, thereby regulating oxygen consumption and limiting the production of excessive reactive oxygen species [60]. Dietary antioxidants may further support these effects, providing a mechanistic explanation for the greater efficacy of whole-food nitrate sources [50,58]. Thus, the nitrite-NO pathway constitutes a redox-sensitive, mitochondria-linked regulatory system that complements NOS-dependent signalling. By enabling the targeted release of NO under hypoxic and metabolic stress conditions, this pathway supports vascular function, mitochondrial efficiency, and metabolic homeostasis, particularly in ageing populations.

### 3.3. Role of Antioxidants in Stabilising NO

Antioxidants such as polyphenols and vitamin C play a key role in maintaining the bioactivity of NO by preventing its oxidative degradation and stabilising NO-derived intermediates. Conditions associated with endothelial dysfunction, including hypercholesterolaemia, diabetes, hypertension, smoking and ageing, are characterised by increased superoxide production and oxidised LDL. These factors rapidly inactivate NO and impair vascular function [61]. The diffusion-limited reaction between NO and superoxide produces peroxynitrite (ONOO^−^), a potent oxidant which reduces NO bioavailability further and promotes oxidative damage [62].

Antioxidant vitamins mitigate these processes. Vitamins C and E reduce superoxide accumulation and lipid peroxidation, thereby preserving endothelial function. Ascorbate also supports NO signalling by maintaining an intracellular redox balance, promoting the formation of S-nitrosothiols (RSNOs) as NO reservoirs, and facilitating nitrite reduction under acidic conditions [61,63]. Crucially, ascorbate helps to maintain tetrahydrobiopterin (BH_4_) in its reduced form, thereby preventing eNOS uncoupling and shifting production from NO to superoxide [64].

**Figure 3:**
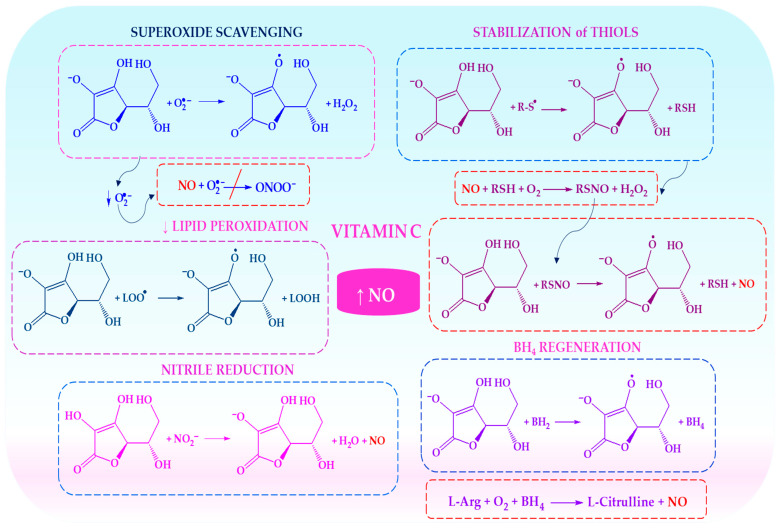
Role of vitamin C in supporting nitric oxide bioavailability. Vitamin C (ascorbate) acts as a water-soluble antioxidant that preserves intracellular thiols and promotes the formation of S-nitrosothiols (RSNO), relatively stable compounds that serve as circulating and intracellular reservoirs of nitric oxide (NO). These molecules can release bioactive NO when required. Ascorbate also supports NO signalling through several complementary mechanisms, including scavenging reactive oxygen species, promoting NO release from RSNO, and reducing nitrite (NO_2_^−^) to NO under acidic conditions. In addition, vitamin C helps maintain tetrahydrobiopterin (BH_4_) in its reduced form, thereby preventing endothelial nitric oxide synthase (eNOS) uncoupling, which otherwise leads to superoxide rather than NO production. Abbreviations: BH_2_—dihydrobiopterin (oxidized form of biopterin); BH_4_—tetrahydrobiopterin, an essential cofactor for nitric oxide synthase; H_2_O_2_—hydrogen peroxide; L-Arg—L-arginine; LOO^•^—lipid peroxyl radical; LOOH—lipid hydroperoxide; NO—nitric oxide; NO_2_^−^—nitrite; O_2_^•−^—superoxide anion radical; ONOO^−^—peroxynitrite; R-S^•^—thiyl radical; RSH—thiol (reduced sulfhydryl group); RSNO—S-nitrosothiol.

Figure 3 shows how vitamin C (ascorbate) increases the bioavailability of NO by acting as an antioxidant. This involves promoting the formation and release of S-nitrosothiols, reducing nitrite to NO in acidic environments and preserving tetrahydrobiopterin to ensure that eNOS functions properly.

Recent studies have extended this concept to whole food matrices that are rich in polyphenols. Diets high in phenolic compounds (e.g., ferulic acid and resveratrol) enhance antioxidant capacity and upregulate endogenous defence systems such as glutathione, catalase and superoxide dismutase. They also reduce lipid peroxidation, thereby creating conditions that favour NO stability [65]. Restoring NO bioavailability in vascular disease therefore requires the provision of NO precursors and the control of oxidative stress [66,67]. Polyphenols may further stabilise NO signalling by modulating NADPH oxidase activity and supporting NO-heme intermediates [68].

Thus, antioxidants regulate NO homeostasis by limiting oxidative degradation, stabilising intermediate reservoirs (e.g., RSNOs), preventing eNOS uncoupling, and promoting nitrite reduction. Consequently, antioxidant-rich diets are integral to strategies aimed at improving vascular and metabolic health, particularly under conditions of oxidative stress and age-related endothelial dysfunction.

### 3.4. Betalains and Flavonoids as Modulators of Oxidative and Inflammatory Pathways

Betalains, particularly betacyanins such as betanin, act as potent modulators of oxidative and inflammatory stress through complementary mechanisms. They directly scavenge reactive oxygen species (ROS), inhibit lipid peroxidation, and activate the Nrf2/ARE pathway. This induces the production of antioxidant enzymes, including heme oxygenase-1, glutathione S-transferase, and NAD(P)H:quinone oxidoreductase-1 [69,70]. This dual activity–direct antioxidant action combined with gene regulation–distinguishes them from conventional antioxidants. They also suppress pro-inflammatory signalling by inhibiting NF-κB and reducing cytokines, thereby protecting cardiovascular and metabolic tissues [71,72]. However, their bioactivity depends on stability within food matrices, as pH, temperature and light exposure can influence degradation and bioavailability [73,74].

Flavonoids, which often co-occur with betalains in nitrate-rich vegetables, reinforce these effects through complementary pathways. They inhibit pro-inflammatory enzymes (COX-2 and iNOS), modulate MAPK signalling, and regulate cytokine profiles by decreasing TNF-α and IL-6, while increasing IL-10 [75,76]. Additionally, flavonoids enhance endothelial function by increasing eNOS activity and decreasing NADPH oxidase-derived superoxide. Beetroot-derived flavonoids exhibit antioxidant and anticancer properties, and they may act synergistically with betalains to reduce oxidative damage [77]. Evidence from exercise models further suggests that interventions rich in betalains attenuate oxidative stress and inflammation, potentially via improved mitochondrial efficiency [78].

These phytochemicals are particularly important for combatting the effects of ageing, where oxidative stress, mitochondrial dysfunction and chronic inflammation can impair vascular integrity. Betalains and flavonoids work together to restore the body’s redox balance, reduce inflammation and support endothelial function [69,71,75]. These phytochemicals are present in foods such as beetroot, Swiss chard and prickly pear, and represent an accessible dietary strategy for enhancing vascular resilience and promoting healthy ageing [77,79].

### 3.5. Potential Enhancement of Nitrate-Derived NO Bioactivity

To enhance nitrate-derived NO bioactivity, it is necessary to optimise each step of the enterosalivary nitrate-nitrite-NO pathway, as well as considering the synergistic interactions with dietary bioactives. Nitrate-rich foods, such as beetroot, are particularly effective as they provide both nitrates and stabilising phytochemicals, such as betalains and polyphenols, which limit oxidative degradation and support endothelial function [80]. Around 25% of circulating nitrate is concentrated in the salivary glands, where it is reduced to nitrite by oral bacteria. This critical step is influenced by diet, the composition of the oral microbiome, and hygiene practices. However, antibacterial mouthwashes can hinder this process by reducing nitrite formation, thereby attenuating vascular benefits [31,81].

Following absorption, nitrite serves as a systemic NO precursor. Red blood cells (RBCs) play a pivotal role in facilitating nitrite reduction via deoxyhaemoglobin, especially in hypoxic and acidic conditions. This enables the targeted delivery of NO to oxygen-deprived tissues [82]. Clinical studies confirm that nitrate supplementation improves endothelial function, reduces blood pressure, and enhances tissue perfusion. There is also potential for benefits in cardiovascular and pregnancy-related conditions [83,84,85]. The effects are generally more pronounced when nitrate is consumed in whole foods rather than in the form of isolated salts, which highlights the importance of the food matrix [13,86].

Additional dietary components can further modulate this pathway. For example, flavonoids enhance endothelial function, reduce oxidative stress and stabilise NO intermediates to improve NO bioavailability, and some also stimulate eNOS activity [87]. Polyphenols and betalains contribute to prolonged NO signalling by protecting against oxidative degradation [80]. Epidemiological and clinical data link the intake of vegetable-derived nitrates with reduced arterial stiffness, lower platelet aggregation, and protection against ischaemia-reperfusion injury. These effects are influenced by dose, microbiome integrity, and redox status [88,89].

RBC integrity also modulates these responses. Alterations in RBC lifespan and membrane properties, as observed in chronic kidney disease, are associated with impaired nitrite reduction and reduced vascular benefits [90]. Therefore, variability in response to nitrate supplementation reflects differences in microbiome composition, redox balance and RBC function. Thus, the bioactivity of nitrate-derived NO is maximised through the combined effects of dietary phytochemicals, oral microbial activity, red blood cell-mediated nitrite reduction, and nitrate consumption in whole food matrices.

## 4. Nitrate-Rich Vegetables: Composition, Phytochemicals and Bioavailability

### 4.1. Beetroot: Composition, Phytochemical Complexity and Functional Food Potential

The functional potential of beetroot (*Beta vulgaris* L.) is attributed to its rich and diverse phytochemical composition, which extends beyond its well-known nitrate content. It contains numerous bioactive compounds, such as the pigments betanin and betaxanthins (betalains), as well as phenolic acids (ferulic, caffeic, gallic and p-coumaric acids), flavonoids (e.g., astragalin, kaempferol, rutin and quercetin), carotenoids, coumarins, terpenoids, alkaloids and tannins [91,92,93,94].

Depending on agronomic and storage conditions, it is also one of the richest dietary sources of inorganic nitrate (250–400 mg/100 g fresh weight) [80,81,82,83,84,85,86,87,88,89,90,91,92,93,94,95]. Betalains, which are nitrogen-containing pigments characteristic of beetroot, exhibit strong antioxidant, anti-inflammatory and cytoprotective properties. Betanin acts as a ROS scavenger and modulates Nrf2-dependent pathways [77,79,96]. These compounds are water-soluble and remain biologically active under physiological conditions [97,98].

Phenolic acids and flavonoids further enhance these effects by inhibiting pro-inflammatory enzymes, modulating NF-κB signalling, and stabilising reactive nitrogen species [99]. The co-occurrence of nitrates with antioxidant phytochemicals is important because these compounds protect NO from oxidative degradation, thereby enhancing its bioavailability and vascular effects. Additionally, beetroot is a source of vitamins (C and folate), minerals (iron, potassium and magnesium) and dietary fibre, as well as betaine, which contributes to homocysteine metabolism and cardiometabolic regulation [100].

**Figure 4:**
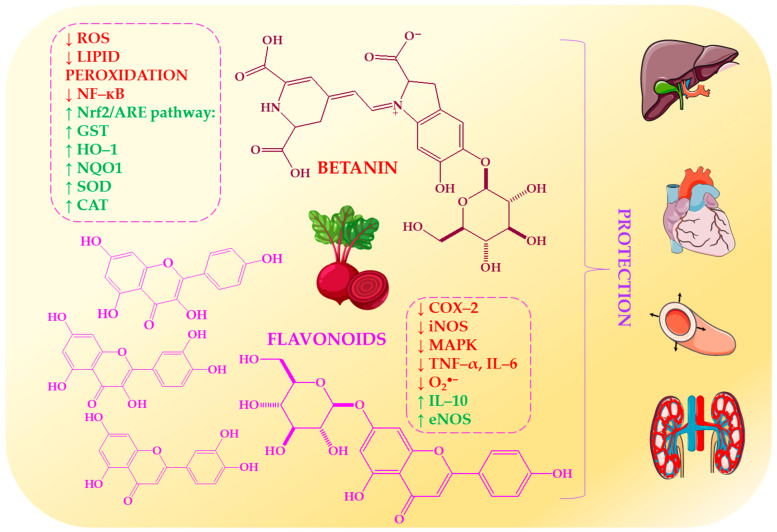
Betalains and flavonoids as multi-pathway modulators of redox and inflammatory signalling. Betanin and other betalains act as multi-pathway modulators that directly scavenge reactive oxygen species (ROS), inhibit lipid peroxidation, and activate the Nrf2/ARE signalling pathway, promoting the expression of antioxidant and detoxifying enzymes such as HO-1, GST, and NQO1. In addition to direct radical scavenging, betalains regulate cellular redox balance and reduce inflammation by suppressing NF-κB signalling and pro-inflammatory mediators. Flavonoids, often co-occurring with betalains in beetroot, further enhance these protective effects by inhibiting inflammatory pathways (COX-2, iNOS, MAPK), modulating cytokine profiles, and improving endothelial function through eNOS activation and reduced NADPH oxidase-derived superoxide. Together, these compounds provide complementary antioxidant and anti-inflammatory actions that support cellular redox homeostasis and nitric oxide bioavailability. Image provided by Servier Medical Art (https://smart.servier.com, accessed on 1 March 2026), licensed under CC BY 4.0 (https://creativecommons.org/licenses/by/4.0/, accessed on 1 March 2026). Abbreviations: ARE—antioxidant response element; CAT—catalase; COX-2—cyclooxygenase-2; eNOS—endothelial nitric oxide synthase; GST—glutathione S-transferase; HO-1—heme oxygenase-1; IL-6—interleukin-6; IL-10—interleukin-10; iNOS—inducible nitric oxide synthase; MAPK—mitogen-activated protein kinase; NF-κB—nuclear factor kappa B; NQO1—NAD(P)H: quinone oxidoreductase 1; Nrf2—nuclear factor erythroid 2—related factor 2; ROS—reactive oxygen species; SOD—superoxide dismutase; O_2_^•−^—superoxide anion radical; TNF-α—tumor necrosis factor alpha.

Figure 4 shows how beetroot’s betalains and flavonoids act as multi-pathway modulators. They scavenge ROS, activate the Nrf2/ARE antioxidant pathways, suppress NF-κB-mediated inflammation and enhance endothelial function. This supports redox balance and nitric oxide bioavailability.

This composition highlights beetroot’s status as a model functional food. Its high nitrate content supports the enterosalivary nitrate-nitrite-NO pathway, thereby improving vascular function and oxygen utilisation [86,93]. A typical serving of beetroot juice (250–500 mL) contains 300–600 mg of nitrates and has been linked to enhanced endothelial function and lower blood pressure [101]. The synergistic interaction between nitrate and other bioactive compounds, such as betalains and polyphenols, further enhances redox balance and anti-inflammatory responses [77,100,102].

Beetroot is widely used in functional food products, including juices, concentrates, powders, and fermented products. The most extensively studied forms are beetroot juice and extracts, which have been shown to improve antioxidant status, vascular function, and exercise performance, partly through enhanced mitochondrial efficiency [101,103]. Fermentation may further increase the stability and bioavailability of phytochemicals [100], while fortified products (e.g., beverages) have demonstrated beneficial metabolic effects [104]. Beetroot is also used as a natural colourant (E162) and a nutraceutical ingredient [96,100].

Its functional relevance is particularly evident in ageing populations. Reduced NOS activity and bioavailability, as well as alterations to the oral microbiome, can impair nitrate metabolism. Meanwhile, oxidative stress accelerates NO degradation [101,105,106,107]. Beetroot phytochemicals can counteract some of these effects by reducing oxidative stress and stabilising NO intermediates [77,86]. However, proton pump inhibitors and antibacterial mouthwashes may reduce nitrate-nitrite-NO conversion [102,108]. As discussed earlier in this review [29,31,33], the impact of oral hygiene practices on nitrate metabolism has been examined. We highlighted that disrupting oral nitrate-reducing bacteria impairs the enterosalivary nitrate–nitrite–NO pathway. Recent evidence further reinforces this concept by showing that broad-spectrum antibacterial mouthwashes markedly reduce the abundance and activity of these bacteria. This leads to lower salivary nitrite formation and diminished NO bioavailability. Beyond its cardiovascular implications, mouthwash-induced oral dysbiosis has also been linked to broader systemic effects, including potential neurocognitive consequences, as suggested by Boulares et al. [108]. These findings emphasise the importance of maintaining an intact oral microbiome and avoiding antiseptic rinses at times when nitrate is being consumed, in order to preserve efficient nitrate reduction and maximise the associated physiological benefits. Thus, beetroot is a multifunctional food in which nitrates, polyphenols and betalains act together to increase NO bioactivity, reduce oxidative stress and promote vascular and metabolic health.

### 4.2. Other Nitrate-Rich Vegetables: Diversity and Comparative Composition

Although beetroot is widely used as a model in dietary nitrate research, many commonly consumed vegetables, particularly leafy and stem varieties, contain comparable or higher levels of nitrates. Vegetables from the *Amaranthaceae*, *Brassicaceae* and *Apiaceae* families typically accumulate high levels of nitrates, including spinach (*Spinacia oleracea*), chard (*Beta vulgaris* subsp. *cicla*), rocket (*Eruca sativa*), mustard (*Brassica juncea*), radish (*Raphanus sativus*), celery (*Apium graveolens*) and parsley (*Petroselinum crispum*) [109,110]. This is largely due to their capacity to store nitrate in vacuoles and their high nitrogen uptake during vegetative growth.

Spinach typically contains 250–500 mg of nitrate per 100 g of fresh weight (occasionally exceeding 700 mg), while rocket may contain 300–600 mg or more per 100 g under intensive cultivation [111,112,113]. Lettuce ranges from 100 to 400 mg/100 g, with higher levels found in varieties grown in greenhouses, while celery generally contains 150–250 mg/100 g [111,112]. By contrast, *Brassica* vegetables (e.g., cabbage, kale, broccoli and cauliflower) typically exhibit moderate nitrate levels (50–250 mg/100 g).

Leafy vegetables are a significant dietary source of nitrate and can contribute considerably to daily intake, particularly in plant-based diets. However, their physiological effects depend not only on nitrate content, but also on phytochemical composition. These vegetables contain a variety of bioactive compounds that affect redox balance and nitric oxide stability. Spinach, for example, is rich in flavonoids (such as spinacetin derivatives), phenolic acids and carotenoids [114,115]. Rocket provides quercetin and kaempferol glycosides, as well as hydroxycinnamic acids [116,117], while lettuce contains chlorogenic acid and flavonoids. Anthocyanins in red cultivars enhance the antioxidant capacity of lettuce [118,119]. Celery contains flavones such as apigenin and luteolin, as well as anti-inflammatory and vasorelaxant coumarins [120,121]. *Brassica* vegetables provide phenolic compounds, vitamins C and E, carotenoids, and glucosinolates [122,123].

Glucosinolates are hydrolysed to isothiocyanates, which have chemoprotective and anti-inflammatory properties and activate Nrf2-dependent antioxidant pathways [124,125]. While they do not directly participate in nitrate reduction, they can indirectly support NO bioactivity by enhancing antioxidant defences and reducing oxidative stress [126,127].

The interaction between nitrates and coexisting antioxidants is a key factor in determining physiological efficacy. Polyphenols and vitamin C can promote the reduction of nitrite to NO under gastric conditions, protecting it from oxidative degradation [113,128]. Therefore, vegetables with a high nitrate and antioxidant content (e.g., spinach and rocket) may increase NO production and stability. Other compounds, such as the anthocyanins found in lettuce and the flavones found in celery, may also support vascular effects. Meanwhile, glucosinolate-derived metabolites found in *Brassica* species contribute to the process via redox-sensitive signalling pathways [129,130,131].

**Figure 5:**
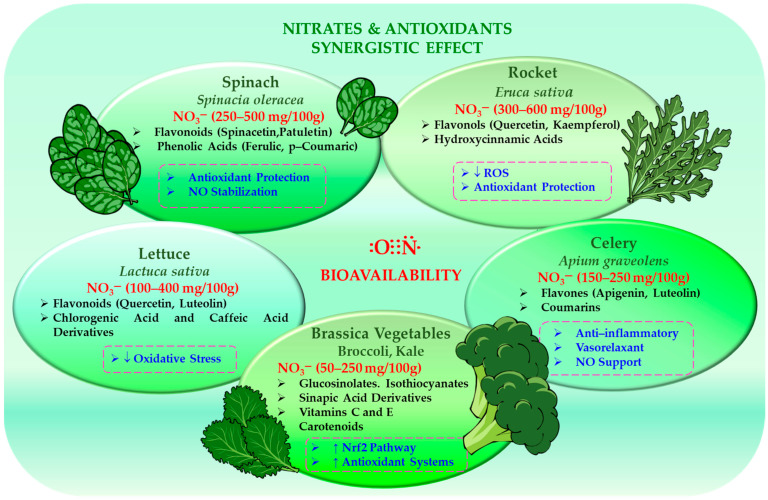
Phytochemical profiles of selected vegetables supporting antioxidant defence and NO stability. Several leafy vegetables contain diverse phytochemicals that contribute to antioxidant defence and may support nitric oxide (NO) stability. Spinacia oleracea (spinach) is rich in flavonoids, phenolic acids, and carotenoids, including spinacetin and patuletin derivatives, ferulic and p-coumaric acids, lutein, and β-carotene. Eruca sativa (rocket) contains quercetin and kaempferol glycosides as well as hydroxycinnamic acids that support radical scavenging and redox signalling. Lactuca sativa (lettuce) provides chlorogenic acid, caffeic acid derivatives, and flavonoids, while red-leaf cultivars also contain anthocyanins that enhance antioxidant potential. Apium graveolens (celery) is characterised by high levels of flavones such as apigenin and luteolin, along with coumarins associated with anti-inflammatory and vasorelaxant effects. Brassica oleracea species contain phenolic compounds, vitamins C and E, and carotenoids, forming a broad antioxidant network. A distinctive feature of Brassica vegetables is the presence of glucosinolates, which are hydrolysed to isothiocyanates that activate Nrf2-dependent antioxidant pathways, indirectly supporting NO bioactivity by strengthening endogenous antioxidant defence. Image provided by Servier Medical Art (https://smart.servier.com, accessed on 1 March 2026), licensed under CC BY 4.0 (https://creativecommons.org/licenses/by/4.0/, accessed on 1 March 2026). Abbreviations: NO—nitric oxide; NO_3_^−^—nitrate; Nrf2—nuclear factor erythroid 2—related factor 2; ROS—reactive oxygen species.

Figure 5 shows the phytochemical composition of selected vegetables, emphasising the presence of compounds such as flavonoids, phenolic acids, carotenoids and glucosinolates, which boost antioxidant defences, support redox signalling and help to maintain the stability and bioavailability of nitric oxide.

Therefore, nitrate concentration alone is insufficient to evaluate functional potential. It is the overall phytochemical matrix—comprising polyphenols, carotenoids, vitamins and, in some cases, glucosinolates—that determines the redox environment in which nitrate-derived NO is generated and maintained. This complexity is essential for understanding the effects of dietary nitrate and for the rational design of functional foods.

## 5. Clinical Evidence in General and Ageing Populations

### 5.1. Blood Pressure and Cardiovascular Outcomes

There is a substantial body of clinical evidence supporting the antihypertensive effects of nitrate-rich vegetables, particularly beetroot and leafy greens, in general and ageing populations. Randomised controlled trials (RCTs) consistently demonstrate that taking nitrate-rich beetroot juice or spinach supplements reduces systolic and diastolic blood pressure, primarily by increasing NO bioavailability via the nitrate-nitrite-NO pathway. This mechanism is particularly important in ageing populations, where eNOS activity is impaired. Across studies, reductions in systolic blood pressure of approximately 3–10 mmHg have been reported, indicating clinically significant cardiovascular benefits (Table 1).

Jonvik et al. [142] provided mechanistic evidence by demonstrating that the ingestion of nitrate-rich vegetables significantly increases plasma nitrate and nitrite concentrations while lowering blood pressure in healthy adults. In their semi-randomised crossover study, spinach was found to induce the highest plasma nitrite levels, while beetroot juice, rocket, and spinach were found to reduce systolic blood pressure (800 mg nitrate from different sources). No such effect was observed with sodium nitrate alone, suggesting that whole food matrices enhance NO bioavailability due to the presence of additional bioactive compounds [142].

These findings are supported by a systematic review of 11 RCTs [143], which concluded that beetroot juice is an effective, low-cost strategy for reducing blood pressure in individuals with normal, pre-hypertensive or hypertensive blood pressure. The effects were mainly attributed to nitrate-derived NO, with additional contributions from secondary metabolites. Narrative reviews similarly highlight beetroot as a non-pharmacological approach to improving vascular function through enhanced NO production, vasodilation, and improved blood flow [144,145].

However, long-term evidence is less consistent. In the DINO3 trial, Li et al. (2024) [146] found that 16 weeks of supplementation with a high-nitrate vegetable powder (approximately 400 mg per day) did not significantly improve office or ambulatory blood pressure, arterial stiffness, or lipid profiles, compared with a low-nitrate control group [146]. These findings suggest that prolonged supplementation may not provide additional benefits beyond habitual vegetable intake for individuals with established hypertension.

Beyond blood pressure, several RCTs have evaluated vascular function markers, including flow-mediated dilation (FMD) and pulse wave velocity (PWV). Meta-analyses indicate that dietary nitrate significantly improves FMD following both acute and chronic intake, reflecting enhanced endothelial function [147,148,149,150]. Acute supplementation with beetroot juice has been shown to increase FMD within hours in older adults [151], while longer-term interventions (e.g., six weeks) have been shown to improve endothelial function and reduce arterial stiffness in hypercholesterolemic individuals [152,153].

Dose-response studies suggest that a daily nitrate intake of around 250–600 mg (4–10 mmol) is sufficient to produce haemodynamic effects, such as reducing blood pressure and improving vascular function [147,148,154]. Acute high doses may produce rapid reductions in blood pressure, whereas regular intake of nitrates from whole vegetables provides sustained vascular benefits and is well tolerated [113,153,155]. However, age-related differences exist: for instance, a meta-analysis of individual participants showed limited effects on 24-h blood pressure variability in older adults, whereas younger individuals exhibited greater reductions in nocturnal systolic blood pressure [156].

The magnitude of the response depends on baseline blood pressure and the composition of the oral microbiota, which is essential for nitrate reduction. Using antibacterial mouthwash can impair this process and reduce the blood-pressure-lowering effects of dietary nitrate [157,158].

In ageing populations, the benefits of dietary nitrate extend beyond reducing blood pressure. Improvements in endothelial function and arterial stiffness–key predictors of cardiovascular risk–suggest a role in both primary and secondary prevention [145,159,160,161]. Further effects have been suggested, such as reduced platelet aggregation and improved microvascular perfusion, but these require confirmation [162,163].

Despite these promising findings, there is limited evidence from long-term trials assessing hard cardiovascular endpoints (e.g., myocardial infarction or stroke), which highlights the need for large, well-designed prospective studies, particularly in ageing populations [164,165].

### 5.2. Cognitive Function and Cerebral Blood Flow

There is emerging evidence that dietary nitrate may support cognitive function by improving cerebral perfusion. NO plays a pivotal role in neurovascular coupling, whereby neuronal activity triggers local vasodilation, thereby increasing cerebral blood flow [166,167]. However, age-related declines in endothelial function and NO bioavailability can impair this process, resulting in reduced cerebral perfusion and cognitive decline [168,169]. Enhancing the nitrate-nitrite-NO pathway through dietary nitrate intake may therefore help to restore neurovascular function and support brain perfusion [170,171].

Clinical studies using functional magnetic resonance imaging (fMRI) and transcranial Doppler ultrasound (TCD) provide mechanistic support for these effects. Nitrate supplementation has been shown to increase cerebral blood flow, particularly in frontal brain regions associated with executive function [169,170]. For instance, a crossover study in adolescents revealed that nitrate-rich beetroot juice increased oxygenated haemoglobin in prefrontal regions during working memory tasks, suggesting enhanced neurovascular responsiveness, though no change in cognitive performance was observed [172]. Similarly, in healthy adults, a single dose of beetroot juice modulated prefrontal blood flow during cognitive tasks and improved performance in serial subtraction, suggesting an acute enhancement of cognitive efficiency under higher task demands [169]. Further evidence indicates that nitrate intake can alter cerebral haemodynamics during physiological stress, including reductions in systolic blood pressure and cerebral augmentation index [173].

These effects may be more pronounced in older adults, who typically exhibit reduced baseline cerebral perfusion and greater vascular dysfunction [169,174]. Acute nitrate supplementation has been associated with improved cerebral blood flow and neurovascular responsiveness in this group, highlighting its potential relevance in addressing age-related cognitive decline. However, findings from longer-term interventions are inconsistent. For example, in a 13-week RCT involving overweight and obese older adults, daily beetroot juice supplementation did not significantly impact cognitive function or prefrontal cerebral blood flow compared to a placebo [175].

Notably, nitrate supplementation has been demonstrated to enhance perfusion in white matter regions susceptible to age-related hypoxia. Using arterial spin labelling MRI, increased blood flow in periventricular white matter was observed in older adults following acute nitrate intake, accompanied by improvements in attention and processing speed [170]. These results imply that NO-mediated improvements in microvascular function could facilitate cognitive processes that depend on white matter integrity [176,177].

Despite these promising mechanistic insights, there is limited evidence for sustained cognitive benefits. Most studies are short-term and involve small groups of participants, and results from longer interventions are inconsistent. Therefore, well-designed, long-term randomised trials are needed to determine whether chronic nitrate intake can meaningfully improve cognitive function and reduce the risk of age-related cognitive decline (Table 2) [178].

Thus, dietary nitrate may contribute to the preservation of cerebrovascular function by enhancing cerebral perfusion and microvascular responsiveness, particularly in ageing populations. If this mechanism is confirmed in long-term studies, nitrate-rich foods could be included in dietary strategies aimed at maintaining cognitive health.

### 5.3. Physical Performance and Sarcopenia

Sarcopenia is defined as the age-related decline in skeletal muscle mass and function. It is associated with impaired muscle perfusion and reduced mitochondrial efficiency, which leads to decreased exercise tolerance and functional capacity [183]. NO plays a central role in regulating muscle blood flow, mitochondrial respiration, and contractile function [184]. Dietary nitrate, which is found in high concentrations in vegetables such as beetroot, increases NO bioavailability via the nitrate-nitrite-NO pathway, particularly in conditions of hypoxia or high metabolic demand, which are characteristic of ageing muscle [41,59,185,186]. This provides a mechanistic basis for potential improvements in muscle function and exercise performance (Table 3).

Clinical evidence shows that nitrate supplementation can improve exercise tolerance, although results are variable. For example, in a randomised crossover study, beetroot juice increased time to exhaustion and reduced central and peripheral fatigue during knee extensor exercise, accompanied by lower perceived effort and muscle pain [197]. However, another study reported increased plasma nitrate/nitrite and reduced blood pressure and blood flow during exercise without improvements in oxygen utilisation, neuromuscular fatigue or performance outcomes. This indicates that physiological effects do not always translate into functional benefits [198].

Short-term nitrate supplementation has been shown to improve muscle oxygenation and reduce the oxygen cost of submaximal exercise, thereby enhancing muscular efficiency [43]. These effects may translate into improved walking economy, muscle power, and tolerance to high-intensity activity–all of which are critical for maintaining independence in older adults [174,182]. Further support for this relationship comes from observational studies, which show that higher habitual nitrate intake is associated with greater handgrip strength, better functional performance, and a reduced risk of mobility impairment and falls in older populations [189,199].

Evidence from systematic reviews and meta-analyses indicates modest but consistent ergogenic effects of dietary nitrate. Supplementation has been associated with improvements in time to exhaustion, muscular endurance, peak power output, and exercise efficiency, particularly at doses of at least 6 mmol/day (approximately 372 mg) and with repeated intake [193,200]. Chronic supplementation appears to be more effective than acute dosing for high-intensity performance, suggesting a potential role in reducing peripheral fatigue [201]. However, the magnitude of the effects varies depending on training status, baseline nitrate intake, and oral microbiota composition [194].

In older adults, nitrate supplementation has been associated with improvements in functional outcomes, such as time to exhaustion, peak power, and the results of mobility tests (e.g., the timed up-and-go and sit-to-stand tests). However, findings are not uniform [174]. The benefits appear to be more pronounced in individuals with reduced NO bioavailability or endothelial dysfunction. Improved muscle perfusion and oxygen delivery may also support anabolic signalling and enhance adaptations to resistance training, suggesting its potential relevance in managing sarcopenia [202,203]. However, not all studies demonstrate performance benefits, particularly in healthy or well-trained individuals. This highlights the influence of study design and inter-individual variability [204]. The current evidence base is limited by the short duration of interventions, small sample sizes, and the heterogeneity of outcomes.

In summary, dietary nitrate supplementation shows promise as an adjunct strategy for supporting muscle perfusion, exercise tolerance, and functional capacity in ageing populations. However, larger, long-term trials are needed to establish whether chronic nitrate intake can meaningfully attenuate sarcopenia, reduce the risk of frailty and improve long-term functional outcomes.

### 5.4. Metabolic Health and Endothelial Dysfunction

Metabolic disorders, including type 2 diabetes mellitus (T2DM), metabolic syndrome, and chronic kidney disease (CKD), are characterised by endothelial dysfunction and reduced NO bioavailability. Hyperglycaemia, insulin resistance and chronic inflammation promote the formation of ROS, which scavenge NO and impair eNOS activity. This accelerates vascular damage and atherogenesis [205,206]. Endothelial dysfunction is now recognised as an early, potentially reversible stage of cardiovascular disease, particularly in ageing populations with metabolic comorbidities [207,208]. In this context, the nitrate-nitrite-NO pathway offers an alternative, oxygen-independent mechanism for generating NO, which can partially restore vascular homeostasis under hypoxic or oxidative conditions [209,210].

Observational studies suggest that a higher intake of dietary nitrates, primarily from plant sources, is associated with improved cardiometabolic profiles. Higher nitrate and nitrite consumption has been linked to lower HbA1c, fasting glucose, blood pressure, and inflammatory markers, as well as increased circulating NO levels [211]. Longitudinal data further indicate that plant-derived nitrate intake is associated with a reduced risk of metabolic syndrome. In contrast, nitrite intake, particularly from animal sources, may have no effect or even have an adverse effect, depending on antioxidant status such as vitamin C intake [212,213]. These findings emphasise the significance of dietary context and the role of co-consumed antioxidants in modulating the metabolic effects of nitrates.

By contrast, evidence from RCTs in people with T2DM remains inconsistent. While short-term nitrate-rich beetroot juice supplementation reliably increases plasma nitrate/nitrite levels, it does not consistently improve blood pressure, endothelial function, or insulin sensitivity [214,215]. However, some studies have reported improvements in secondary outcomes, including reduced inflammatory markers (IL-6, TNF-α and NF-κB) and enhanced cognitive performance. This suggests that there may be pleiotropic effects beyond vascular endpoints [214,215,216].

More consistent vascular benefits have been observed in individuals with other cardiometabolic risk factors. In patients with hypercholesterolaemia, chronic nitrate supplementation has been shown to improve endothelial function (e.g., increased flow-mediated dilation), with modest effects on arterial stiffness and platelet activation [152]. These effects are partly mediated by the enterosalivary nitrate-nitrite-NO pathway and may involve changes in the oral microbiome, including an increased abundance of nitrate-reducing bacteria.

Evidence from paediatric populations suggests a more complex, potentially dose- and source-dependent, relationship. While total nitrate intake is not consistently associated with metabolic syndrome risk, higher nitrite intake has been linked to adverse glycaemic outcomes. Furthermore, both plant- and animal-derived nitrate intake have been associated with elevated blood pressure or triglycerides in certain groups [217]. These findings emphasise the importance of considering both quantity and dietary source when evaluating the early-life effects on the cardiovascular system.

In CKD, reduced NO bioavailability and increased oxidative stress contribute to vascular dysfunction and elevated cardiovascular risk [218]. Preliminary clinical studies suggest that dietary nitrate may improve exercise tolerance and vascular parameters. Acute nitrate supplementation has been shown to increase NO metabolites and enhance exercise efficiency without affecting maximal aerobic capacity [219]. Similarly, small-scale studies have reported reductions in blood pressure and renal resistance index following nitrate intake. However, the effects on renal function markers (e.g., eGFR and albuminuria) remain inconclusive [220,221]. Altered nitrate handling in CKD, as indicated by elevated plasma nitrate levels alongside declining eGFR, highlights the necessity for population-specific dosing strategies [222].

The current evidence suggests that dietary nitrate, primarily sourced from vegetables such as beetroot and leafy greens, may partially restore NO bioavailability and enhance endothelial function in individuals with metabolic disorders (Table 4). While metabolic outcomes such as glycaemic control remain variable, vascular benefits are more consistently observed. Given the central role of endothelial dysfunction in T2DM, metabolic syndrome and CKD, nitrate-rich diets may represent a valuable adjunct to conventional therapies.

While metabolic outcomes such as glycaemic control remain variable, vascular benefits are more consistently observed. Given the central role of endothelial dysfunction in T2DM, metabolic syndrome and CKD, nitrate-rich diets may represent a valuable adjunct to conventional therapies. However, larger, longer-term RCTs are required to clarify their impact on clinically relevant endpoints.

## 6. Age-Related Changes in Nitrate Metabolism

Ageing markedly alters NO homeostasis, reducing the efficiency of the nitrate-nitrite-NO pathway via endothelial dysfunction, oxidative stress and chronic low-grade inflammation (‘inflammageing’) [230]. Although dietary nitrate provides an independent source of NO via nitrite reduction, the bioactivation process is affected by age-related changes in endothelial function, oral microbiota, gastric acidity and systemic redox balance [18]. The enterosalivary cycle, in which approximately 25% of circulating nitrate is concentrated in saliva and converted to nitrite, represents a critical regulatory step that may become less efficient with age [17,231].

Ageing is associated with reduced eNOS activity, increased arginase activity, and enhanced oxidative degradation of NO, which leads to peroxynitrite formation and endothelial damage [168,232]. Consequently, the nitrate-nitrite-NO pathway becomes more important, particularly under hypoxic or acidic conditions [233,234]. However, the effectiveness of this pathway may be limited by alterations to the oral microbiome, reduced salivary flow, chronic diseases, polypharmacy and declining renal function, which affect nitrate clearance [23,222,235,236,237]. These factors contribute to interindividual variability and necessitate personalised approaches to nitrate intake in older adults.

**Figure 6:**
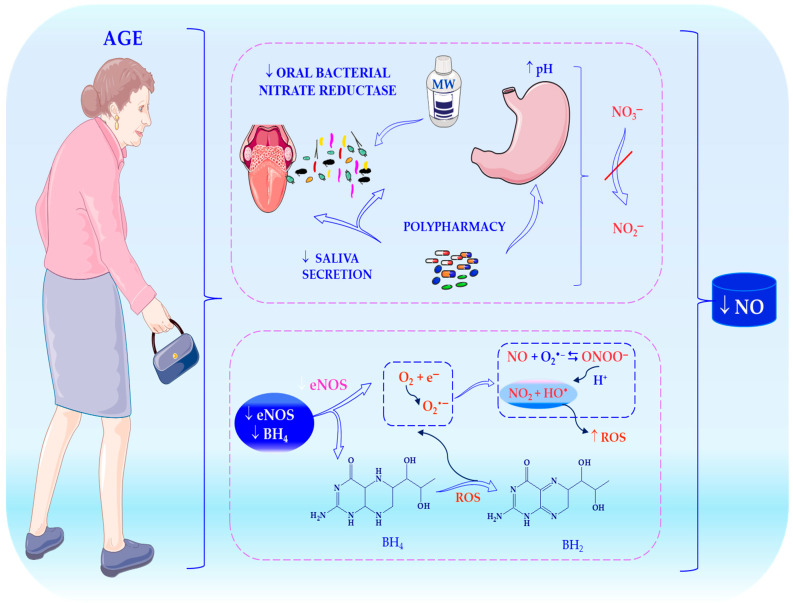
Age-related mechanisms leading to reduced bioavailability of nitric oxide in the vascular system. Vascular aging is associated with reduced expression and activity of endothelial nitric oxide synthase (eNOS), which produces endogenous NO. Functional changes within the endothelium, including impaired shear-stress–induced mechanotransduction and decreased eNOS phosphorylation at its activating site, lead to diminished NO synthesis. At the same time, increased oxidative stress limits the bioavailability of tetrahydrobiopterin (BH_4_), a key eNOS cofactor. BH_4_ deficiency promotes eNOS uncoupling, leading to the enzyme producing superoxide rather than NO. With aging, disturbances also occur in the nitrate–nitrite–NO pathway. The reduction of nitrates to nitrites depends on commensal bacteria located on the dorsal surface of the tongue, and this process may be impaired by age-related changes in the oral microbiome, poorer oral health, decreased salivary secretion, and frequent use of antibacterial mouthwashes. Polypharmacy, common in older adults, may further exacerbate these disturbances, as some medications can alter the oral microbiota or increase oxidative stress, thereby reducing NO bioavailability. Image provided by Servier Medical Art (https://smart.servier.com, accessed on 1 March 2026), licensed under CC BY 4.0 (https://creativecommons.org/licenses/by/4.0/, accessed on 1 March 2026). Abbreviations: BH_2_—dihydrobiopterin; BH_4_—tetrahydrobiopterin; eNOS—endothelial nitric oxide synthase; HO^•^—hydroxyl radical; MW—mouthwash; NO—nitric oxide; NO_2_—nitrogen dioxide; NO_2_^−^—nitrite; NO_3_^−^—nitrate; O_2_^•−^—superoxide anion radical; ONOO^−^—peroxynitrite; pH—potential of hydrogen; ROS—reactive oxygen species.

Figure 6 shows how vascular ageing reduces the bioavailability of NO through several mechanisms: impaired expression and activity of endothelial nitric oxide synthase (eNOS), BH_4_ deficiency causing eNOS uncoupling, increased oxidative stress and impaired nitrate-nitrite-NO conversion due to age-related changes in oral microbiota, salivary secretion, oral health and polypharmacy.

### 6.1. Reduced eNOS Activity and NO Bioavailability

A defining feature of vascular ageing is the reduced expression and activity of eNOS, which is driven by impaired shear stress signalling and decreased phosphorylation at activation sites (e.g., Ser-1177) [238,239]. Concurrent BH_4_ depletion promotes eNOS uncoupling and superoxide production, thereby exacerbating endothelial dysfunction [240,241]. Reduced NO bioavailability leads to impaired vasodilation, increased arterial stiffness and an elevated risk of cardiovascular disease [207,242]. Dietary nitrate provides an alternative NO source, independent of eNOS, under hypoxic or acidic conditions [23,243]. This compensatory pathway may be particularly important for older adults; however, structural vascular changes (e.g., intimal thickening and reduced capillary density) can restrict NO signalling in advanced disease [168,244]. Nevertheless, clinical studies suggest that older individuals may experience substantial improvements in blood pressure and endothelial function after consuming nitrates, possibly due to greater responsiveness resulting from baseline NO deficiency [112,155,245].

### 6.2. Alterations in Oral Microbiota

The reduction of nitrate to nitrite is dependent on oral bacteria located on the dorsal tongue [31,81]. Ageing is associated with reduced microbial diversity and decreased abundance of nitrate-reducing genera (e.g., *Neisseria*, *Rothia* and *Actinomyces*), which could limit NO bioactivation [32]. Hyposalivation, which is common in older adults due to glandular atrophy or medication use, further impairs the concentration and reduction of nitrates in the oral cavity [246,247,248]. Furthermore, periodontal disease, denture use and antiseptic mouthwashes can disrupt the balance of microbes and reduce nitrite formation, thereby reducing the cardiovascular benefits of dietary nitrate [249,250,251,252]. Dietary patterns rich in plant-based foods may support nitrate-reducing microbiota, whereas antimicrobial exposure may impair them. This suggests the potential for microbiome-targeted interventions [249,252].

### 6.3. Increased Oxidative Stress and Endothelial Impairment

Ageing is characterised by increased production of reactive oxygen species (ROS) due to mitochondrial dysfunction and inflammageing, alongside reduced antioxidant capacity [253,254,255]. Superoxide reacts with NO to form peroxynitrite, thereby reducing NO bioavailability and impairing endothelial function [244]. While dietary nitrate can increase NO production, a high oxidative burden may reduce its effectiveness by accelerating NO degradation [128,234,256]. Crucially, nitrate-rich vegetables also contain antioxidants (e.g., vitamin C, polyphenols and betalains) that preserve NO bioactivity and improve endothelial function [86,101,145]. Therefore, whole foods may be more effective than isolated nitrate supplementation. Diets such as the Mediterranean diet or the DASH diet may further enhance these effects [257].

### 6.4. Red Blood Cell Dysfunction and NO Export

Red blood cells contribute to NO bioactivity by reducing nitrite to NO under hypoxic conditions [258,259]. However, ageing can impair RBC deformability, redox balance and haemoglobin function, thereby reducing nitrite reductase activity and NO bioavailability [255,260,261,262]. Altered RBC structure and signalling may also restrict the export of NO to vascular tissues, thereby reducing vasodilatory responses despite adequate nitrite levels [56,128,263]. These changes may contribute to impaired microcirculation, reduced exercise capacity and cognitive decline [264]. Lifestyle interventions, including aerobic exercise and antioxidant-rich diets, may improve RBC function and enhance nitrate efficacy [265,266].

### 6.5. Drug-Nutrient Interactions

Polypharmacy significantly affects nitrate metabolism in older adults [267]. Proton pump inhibitors increase gastric pH, thereby reducing the non-enzymatic conversion of nitrite to NO in the stomach [268,269]. Antibacterial mouthwashes and antibiotics can disrupt nitrate-reducing microbiota, thereby reducing nitrite availability and attenuating blood pressure responses [270,271]. Other medications, including xanthine oxidase (XOR) inhibitors (e.g., allopurinol) and drugs that affect salivary flow or gastrointestinal motility, may further modify nitrate bioactivation [17,272]. These interactions likely contribute to variability in clinical responses. Therefore, consideration of medication use, oral health, and microbiome status is essential for optimising nitrate-based interventions in ageing populations.

Although there is no formal protocol for dietary nitrate intake, there are several practical guidelines that can enhance nitrate reduction and NO bioavailability [29,31,33,108]. These include avoiding antibacterial mouthwashes around the time of consumption, ensuring adequate salivary flow and consuming nitrate-rich beetroot products 1–2 h prior to the desired physiological effect. Minimally processed forms, such as juices, purées, baked or fermented beetroot, and standardised extracts, provide more consistent nitrate delivery and support efficient enterosalivary conversion.

Currently, there are no formal guidelines that specify age- or disease-dependent restrictions for dietary nitrate intake. However, individual responses may be influenced by several physiological factors. Older adults and individuals with chronic conditions associated with reduced nitric oxide signalling or elevated oxidative stress may benefit from beetroot products that preserve both nitrates and supportive phytochemicals. Conversely, gastrointestinal disorders that affect gastric acidity, motility, or the composition of the microbiota may reduce the efficiency of nitrate–nitrite conversion, potentially diminishing the expected physiological effects. These considerations emphasise the importance of personalised approaches for populations with impaired oral, vascular or gastrointestinal function, despite the fact that dietary nitrate from vegetables is generally considered safe when consumed as part of a normal diet.

## 7. Safety, Controversies and Regulatory Aspects

### 7.1. Nitrate vs. Nitrosamine Debate

Safety concerns regarding dietary nitrate primarily relate to its potential conversion into nitrosamines, also known as *N*-nitroso compounds (NOCs) [185,188]. These compounds can be formed when nitrite reacts with amines in acidic conditions or during high-temperature processing, particularly in protein-rich foods such as processed meats [273,274,275]. Epidemiological evidence suggests that consuming large quantities of processed meat increases the risk of developing colorectal cancer, likely due to the formation of NOCs and other factors, such as haem iron [276,277]. However, nitrate itself is not considered to be directly carcinogenic, and its health effects depend strongly on the dietary source. Nitrate from vegetables, which represent the main dietary source, has not been associated with an increased cancer risk and may even demonstrate protective properties in meta-analyses [278,279,280]. This is attributed to the presence of antioxidants (e.g., vitamin C and polyphenols), which inhibit nitrosamine formation and promote the reduction of nitrite to NO.

Beyond nitrosamine formation, nitrate and nitrite have also historically been associated with potential toxic effects, including the risk of methaemoglobinaemia [278,279,280]. This condition results from the oxidation of ferrous haemoglobin to ferric methemoglobin, which is unable to bind oxygen efficiently. Although it is primarily clinically relevant in infants and in cases of contaminated water exposure, methemoglobinemia remains an important toxicological consideration in discussions of nitrate safety [278]. Notably, vegetable-derived nitrate is accompanied by antioxidants that counteract nitrosative stress and limit methaemoglobin formation, which distinguishes plant sources from processed meats [276,277].

Another relevant aspect of nitrate safety is its interaction with nitric oxide-donating drugs, as dietary nitrate can theoretically enhance the vasodilatory effects of pharmacological NO donors by providing additional nitrite-derived NO [29,31,33,108]. While this interaction is generally considered beneficial for people with endothelial dysfunction, individuals receiving nitrate-based medications for cardiovascular conditions should be aware of it. This topic is becoming increasingly recognised in the context of personalised nutrition and pharmacology.

Therefore, the health impact of nitrate intake depends on the food matrix: whole plant foods provide protective co-nutrients, whereas processed meats lack these factors and introduce additional risks such as sodium, saturated fat, and haem iron [281,282,283]. This distinction highlights the potential benefits of dietary nitrate from vegetables for cardiovascular health [284].

### 7.2. Acceptable Daily Intake and Risk Assessment

The Acceptable Daily Intake (ADI) for nitrates, as set by the FAO/WHO and the EFSA, is 3.7 mg per kg of body weight per day. This represents a conservative safety threshold for lifetime exposure. While most people remain within safe limits, some individuals may exceed the ADI if they consume large amounts of nitrate-rich vegetables or are exposed to high levels of nitrate in the environment [285,286]. Occasional exceedance of the ADI is not necessarily associated with adverse effects, particularly when nitrates originate from antioxidant-rich plant foods that limit nitrosation reactions [285]. Risk assessments also identify vulnerable groups, such as infants, who are susceptible to methaemoglobinaemia due to immature haemoglobin metabolism [287].

However, current regulatory frameworks do not fully account for several important factors. One such factor is the influence of antibiotics and antimicrobial agents on nitrate metabolism, as antibiotics can disrupt the oral and gut microbiota responsible for reducing nitrate to nitrite, thereby impairing endogenous NO generation [106,252,271]. This effect has been documented for broad-spectrum antibiotics and antiseptic mouthwashes, both of which can significantly diminish nitrate-reducing bacterial populations. Furthermore, foods themselves—both plant- and animal-derived—contain natural antimicrobial compounds, including polyphenols, glucosinolates, peptides, and in some cases, antibiotic residues [9,243]. These substances may further modulate microbial nitrate reduction, either inhibiting it or reshaping the composition of nitrate-reducing communities.

Such interactions highlight the need for risk evaluation models that are more nuanced and consider not only dose and source, but also microbiome integrity, dietary antimicrobial exposure, and potential interactions with NO-donating drugs [288]. Incorporating these factors is crucial for accurately evaluating the long-term cardiometabolic and cognitive consequences of dietary nitrate consumption.

## 8. Practical Applications and Perspectives

### 8.1. Dietary Nitrate Intake and Its Relevance for Ageing Populations

Increasing dietary nitrate intake may support vascular health in older adults by counteracting the age-related reduction in NO production. Clinical studies indicate that regularly consuming ~250–300 g/day of nitrate-rich vegetables can reduce middle-aged and older individuals’ systolic blood pressure, providing sustained cardiovascular benefits [289]. Systematic reviews suggest improvements in cardiovascular and physical performance; however, the effects on cognition are inconsistent and dependent on dosage [174].

Whole food sources should be prioritised over supplements, since vegetables provide antioxidants and bioactive compounds that enhance NO bioavailability and inhibit nitrosamine formation [185,188]. Importantly, the efficacy of nitrate-rich foods in aged individuals is also influenced by the degree of food processing. Fresh and minimally processed vegetables retain not only their native nitrate content but also vitamin C, betalains and flavonoids, which stabilise nitrite, limit oxidative degradation of NO and support S-nitrosothiol formation, mechanisms that are particularly relevant in older adults with reduced endogenous antioxidant capacity and impaired NO signalling [185,288]. In contrast, highly processed nitrate-containing foods often undergo thermal treatment or prolonged storage, which diminishes phytochemical content and may reduce nitrate stability [210,215,288]. These changes weaken the protective redox environment required for efficient conversion of nitrates to nitrites to NO, which is particularly important in ageing populations characterised by elevated oxidative stress, reduced gastric acidity, and alterations in the oral microbiota that compromise the enterosalivary nitrate cycle [289]. Therefore, consuming fresh or minimally processed nitrate-rich vegetables is likely to provide older adults with superior physiological benefits compared with consuming highly processed products.

However, potential interactions with medications, such as antihypertensives and phosphodiesterase (PDE) inhibitors, should be considered, as these can increase NO-mediated vasodilation [290]. Personalised recommendations are essential and should take into account factors such as oral health, microbiome status, medication use and dietary habits. For example, avoiding antibacterial mouthwashes can help preserve nitrate-reducing oral bacteria and improve physiological responses [291]. Aligning nitrate intake with plant-based or Mediterranean-style dietary patterns may further enhance the benefits.

It has been demonstrated that the timing of optimal intake may affect the efficiency of oral bacterial nitrate reduction [106,252,271,292]. Although liquid beetroot-based supplements are often assumed to have a short oral ‘hang time’, recent evidence shows that beetroot juice and vegetable extracts dissolved in water can produce a significant increase in salivary nitrate within the first 10–30 min after ingestion. This early increase, which has also been observed in controlled experiments using RedNite and NutriSpain extracts, suggests that nitrate may be retained within oral biofilms, released gradually from plant particles or rapidly recirculated through the highly vascularised oral mucosa. Notably, substantial inter- and intra-individual variability in nitrate recycling and nitrite production has been documented, indicating that timing may affect individuals differently. Overall, consuming nitrate-rich foods 1–2 h before the desired physiological effect appears to promote more efficient nitrite formation, especially when salivary flow and oral microbiota are maintained.

The efficacy of beetroot also depends on its form. Fresh and minimally processed products, such as juices, purées, baked or fermented beetroot, retain nitrates and phytochemicals that stabilise nitrite and enhance nitric oxide bioavailability. Concentrated juices and freeze-dried powders provide standardised nitrate doses and are commonly used in clinical settings. However, highly processed beetroot products may exhibit reduced nitrate stability and diminished physiological impact [106,252,271,292].

The growing interest in nitrates has stimulated the development of functional foods, including beetroot-based products and nitrate-enriched formulations designed to deliver effective doses [9,243]. Nevertheless, product design must prioritise safety and regulatory compliance, especially for vulnerable groups such as infants, pregnant women and individuals with impaired renal function [174,284]. Future research should focus on long-term trials to establish the optimal dosage, identify individuals who respond well to treatment and clarify the impact of food matrix effects and microbiome interactions. Incorporating these findings into evidence-based dietary strategies will be crucial to maximising the health benefits of nitrates for ageing populations.

### 8.2. Gastrointestinal and Food Matrix Factors Influencing Nitrate Metabolism

It has been demonstrated that acute and chronic gastrointestinal diseases can substantially alter the metabolism of nitrates and other oxo-nitrogen derivatives by modifying gastric acidity, mucosal integrity, and microbial composition [293]. Conditions such as gastritis, inflammatory bowel disease, coeliac disease and chronic dysbiosis can disrupt the enterosalivary nitrate cycle by decreasing the effectiveness of nitrate reduction to nitrite and the subsequent formation of NO [294]. Altered gastric pH can affect nitrosation reactions and the stability of nitrite-derived NO, while inflammation-associated changes in epithelial permeability can alter nitrate absorption dynamics. Furthermore, gastrointestinal disorders frequently disrupt the oral and intestinal microbiota, reducing populations of nitrate-reducing bacteria and consequently reducing endogenous NO production [295]. These disease-related alterations highlight the importance of considering gastrointestinal health when evaluating nitrate metabolism and its physiological consequences.

Importantly, the indigestible food matrix—particularly the content and composition of dietary fibre—plays a significant role in modulating the absorption and metabolism of nitrates and other oxo-nitrogen derivatives in the gastrointestinal tract [296,297,298]. Soluble fibres can influence gastric emptying and intestinal transit time, potentially altering the kinetics of nitrate release and uptake [293,294,295]. Insoluble fibres and complex plant cell wall structures may entrap nitrate within the matrix, delaying its bioaccessibility until later stages of digestion. Fermentable fibres also influence the composition and activity of the gut microbiota, including nitrate-reducing species. This affects the conversion of nitrate to nitrite and subsequent NO metabolites [185,188,210]. Together, these interactions demonstrate that nitrate bioavailability is not solely determined by its concentration in food, but also by the structural and physicochemical properties of the accompanying plant matrix.

## 9. Limitations and Future Directions

Despite the growing body of evidence supporting the health benefits of nitrate-rich vegetables for vascular and metabolic health, there are still several limitations. Many clinical studies are characterised by small sample sizes, short durations and heterogeneous populations, which limits generalisability. Variability in responses to nitrate supplementation across different age groups highlights the need for stratified analyses, which are not consistently applied [21,24].

Interindividual differences in the oral microbiome, which is critical for nitrate-to-nitrite conversion, represent another underexplored source of variability. Diet, oral hygiene and antibacterial mouthwash use are factors that can significantly affect nitrate bioactivation, yet they are rarely standardised across studies [31,33]. Furthermore, most trials focus on surrogate endpoints (e.g., blood pressure and endothelial function), while long-term clinical outcomes such as cardiovascular events, cognitive decline and mortality remain insufficiently investigated.

The mechanistic interactions between nitrate-derived NO and co-existing phytochemicals (e.g., betalains and flavonoids) also require further clarification. Although synergistic effects have been suggested, the underlying pathways and dose-response relationships are not fully understood [13,80]. Furthermore, the variability of nitrate content in different foods, the various processing methods used, and the potential formation of nitrosative by-products make it difficult to develop consistent dietary recommendations [11,15].

Future research should prioritise well-designed, long-term randomised controlled trials with standardised interventions and integrated assessments of NO bioavailability, gut microbiota composition, and oxidative stress. Multi-omic approaches (e.g., metabolomics, microbiomics and nutrigenomics) could help to identify responders and predictive biomarkers. Further studies are also needed to evaluate cognitive and vascular outcomes in ageing populations, particularly individuals with impaired NO synthesis, in order to facilitate clinical translation.

## 10. Conclusions

Nitrate-rich vegetables are valuable dietary components that enhance nitric oxide (NO) bioavailability and support vascular, metabolic and cognitive function, particularly in ageing populations. These effects arise from nitrate-derived NO and the accompanying phytochemicals that stabilise nitrite and modulate oxidative and inflammatory pathways. Through the enterosalivary nitrate–nitrite–NO pathway and red blood cell-mediated nitrite reduction, these foods augment endogenous NO production, particularly in conditions of hypoxia, metabolic stress or age-related endothelial decline.

Despite inter-individual variability driven by oral microbiota, red blood cell function and dietary context, beetroot and other nitrate-rich vegetables show consistent benefits for muscle performance, cerebral perfusion and cardiometabolic health. Current evidence favours whole-food sources over isolated nitrate salts; however, heterogeneity across studies highlights the need for long-term trials to define optimal intake, assess safety in vulnerable groups, and clarify microbiota-dependent differences in nitrate metabolism. This review highlights key factors influencing nitrate efficacy, such as the timing of intake, food form and processing, the impact of oral hygiene practices and individual physiological status. This integrated perspective will help to refine future recommendations and maximise the therapeutic potential of nitrate-rich vegetables.

## Figures and Tables

**Table 1:** Some studies on the relationship between dietary nitrate and cardiovascular outcomes.

Study Type	Intervention/Exposure	Main Findings	Author/Source
RCT	Flavonoid-rich apples and nitrate-rich spinach	Increased NO status (RXNO, nitrite), improved endothelial function, lower systolic BP	[132]
RCT	Nitrate-rich spinach meal	Acute increase in salivary nitrite/nitrate; reduced systolic BP and pulse pressure; improved large artery elasticity	[133]
RCT	7-day high-nitrate vs. low-nitrate diet	Increased plasma/salivary nitrate and nitrite; no significant change in BP or arterial stiffness	[134]
RCT	1-week beetroot juice (high nitrate) vs. placebo	3–8× increase in nitrate/nitrite levels; no BP change in treated hypertensives	[135]
Prospective cohort	Nitrate intake from vegetables in older women	Higher nitrate intake associated with lower ASVD and all-cause mortality (attenuated after diet adjustment)	[136]
Prospective cohort	Habitual vegetable nitrate intake in women	No independent association with CHD risk after adjusting for lifestyle/diet	[137]
RCT	Nitrate-rich vegetables (~400 mg/day) or beetroot juice	Increased plasma nitrate/nitrite; post-meal BP reduction; diet and juice equally effective short-term	[138]
RCT	12-week high-nitrate vegetable diet or beetroot juice	Systolic BP reduction in vegetable group; no change in juice or diastolic BP	[139]
RCT	Beetroot juice + vitamin C vs. beetroot juice alone	Lower daily systolic BP; higher urinary/salivary nitrate/nitrite; vitamin C enhances vascular protection	[140]
Systematic review	Habitual vegetable nitrate intake	Inverse association with CVD risk/mortality; risk reduction plateaus at moderate intake	[141]

**Table 2:** Some studies on the effects of beetroot juice and dietary nitrate supplementation on physical performance, vascular health and cognitive function.

Study Type	Intervention/Exposure	Main Findings	Author/Source
RCT	Healthy adults; flavonoid-rich apples, nitrate-rich spinach (acute)	Increased NO status; no acute improvement in cognitive function or mood	[179]
RCT	Older (50–70 y) vs. younger adults; acute BRJ (10.5 mmol nitrate)	Increased plasma nitrate/nitrite; decreased systolic BP in both, diastolic BP more in older adults; cognitive reaction time improved	[21]
RCT	Overweight/obese older adults; 13-week incremental BRJ doses	Feasible long-term supplementation; dose-dependent increase in plasma and urinary nitrate	[180]
RCT	Overweight/obese middle-aged/older adults; CR ± high-nitrate BRJ (14 days)	Microvascular and cognitive function improved more with CR + BRJ; BP and body composition unchanged	[181]
Review	Humans and preclinical models	Nitrate may support cognitive health via NO-dependent neurovascular coupling; effects depend on intervention length and participant health	[177]
Systematic review	Healthy adults and athletes	BRJ improves endurance, oxygen efficiency, muscular power; evidence for cognitive benefits is mixed; potential role in healthy aging	[182]

**Table 3:** Some studies on the effects of dietary nitrate and beetroot juice on physical performance.

Study Type	Population/Intervention/Exposure	Main Findings	Author/Source
RCT	9 healthy adults, sodium nitrate 0.1 mmol/kg/day	Reduced VO_2_max but trend to increased time to exhaustion; improved muscle energetic efficiency	[187]
Review	Humans (various), dietary nitrate (mainly beetroot juice)	Improves muscle efficiency and contractile function; reduces O_2_ cost of submaximal exercise	[188]
Cross-sectional	1420 older women (≥70 y), habitual dietary nitrate	Higher nitrate intake associated with stronger grip strength and faster TUG; lower odds of weakness and slow function	[189]
RCT	15 active males, BRJ 140 mL/d (985 mg/d)	No improvement in maximal strength, CMJ, or muscular endurance; slight increase in knee flexion power at 60°·s^−1^	[190]
RCT	8 endurance-trained males, BRJ short-term supplementation	No effect on high-intensity intermittent running in normoxia or hypoxia	[191]
RCT	9 professional tennis players, acute BRJ 70 mL (6.4 mmol NO_3_^−^)	No improvement in match-play running performance, serve speed, or handgrip	[192]
Meta-analysis	123 RCTs, 1705 adults, nitrate via BRJ, salts, or diet	Improved exercise performance 2–10 min; acute 5–14.9 mmol ≥ 150 min prior optimal; oral microbiota influences effect	[193]
RCT	15 elite football players, BRJ 400 mg NO_3_^−^	Supplementation effective only if habitual nitrate intake <300 mg; no effect above	[194]
RCT	80 winter triathletes, BRJ 6.5 mmol NO_3_^−^/70 mL × 3/day for 7 d	Improved high-speed running economy and cycling TTE; no effect on 10-km XC skiing	[195]
RCT	14 postmenopausal women with hypertension, acute BRJ 800 mg and 1-week 400 mg/d	Reduced post-exercise SBP; improved FMD and parasympathetic HRV recovery	[196]
Umbrella review	Professional athletes and healthy adults, BRJ nitrate-rich	Small improvements: muscle strength in athletes, aerobic endurance in non-athletes; acute and chronic supplementation effective	[49]
Systematic review	Adults and athletes, BRJ nitrate-rich	Strong potential to enhance physical performance; cognitive benefits inconclusive	[182]

**Table 4:** Selected studies on the effects of dietary nitrate on metabolic health and endothelial dysfunction.

Study Type	Population/Intervention/Exposure	Main Findings	Author/Source
RCT	30 healthy men and women, flavonoid-rich apples and nitrate-rich spinach (acute)	Increased plasma NO species, improved endothelial function, reduced systolic BP; no additive effect for combination	[132]
Review	Humans and preclinical, dietary nitrate via vegetables and beetroot	Nitrate improves BP, endothelial function, exercise performance; protects against ischemia-reperfusion injury; effects depend on diet and nutrients	[113]
RCT	48 T2DM adults, NR beetroot juice 70 mL/day, 6.43 mmol nitrate/day for 4 days	Increased plasma nitrate/nitrite, but no effect on O_2_ cost of walking or 6MWT performance	[215]
RCT	69 hypercholesterolemic adults, NR beetroot juice daily for 6 weeks	Improved FMD (∼24%), small decrease in PWV, reduced platelet-monocyte aggregates; altered oral microbiome	[152]
Observational	1546 adults (Tehran Lipid and Glucose Study), Dietary nitrate-containing vegetables	Higher NCV intake associated with lower baseline eGFR and higher CKD prevalence; no association with 3-year CKD incidence	[223]
RCT	23 adults with Raynaud’s phenomenon, NR beetroot juice acute and 14-day supplementation	Improved peripheral blood flow, endothelial function, anti-inflammatory status, reduced BP; plasma nitrite increased	[224]
RCT/Commentary	General population under COVID-19 confinement, Nitrate-rich vegetables (beetroot)	Recommended to mitigate endothelial dysfunction due to inactivity, stress, unhealthy diet; potential cardiovascular protection	[225]
RCT	13 HIV-infected and 18 healthy adults, NR beetroot juice acute	Improved %FMD in both HIV and healthy adults; no PWV changes	[226]
Review	Humans, dietary nitrate supplementation	Explored nitrate-nitrite-NO pathway; enhances NO bioavailability, skeletal muscle function, exercise performance, vascular health	[185]
RCT	12 late-postmenopausal females, NR beetroot juice single dose 140 mL, 600 mg NO_3_^−^	Improved resting macrovascular function and resistance to ischemia-reperfusion injury; postmenopausal stage-dependent effects	[227]
RCT	12 postmenopausal females, NR beetroot juice acute 140 mL	Reduced resting and exercise mean arterial pressure; no changes in flow-mediated vasodilation; effective for BP management	[228]
RCT	19 adults with WHO Group 3 pulmonary hypertension, NR beetroot juice 140 mL	Improved endurance shuttle walk time, FMD, lowered mean arterial pressure	[229]

## Data Availability

No new data were created or analyzed in this study. Data sharing is not applicable to this article.
